# An international survey of the structure and process of care for traumatic spinal cord injury in acute and rehabilitation facilities: lessons learned from a pilot study

**DOI:** 10.1186/s12913-022-08847-w

**Published:** 2022-12-21

**Authors:** Aidin Abedi, Fin Biering-Sørensen, Harvinder S. Chhabra, Julia Maria D’Andréa Greve, Nasser M. Khan, Eerika Koskinen, Kenny Yat Hong Kwan, Nan Liu, James W. Middleton, Sasa Moslavac, Vafa Rahimi-Movaghar, Colleen O’Connell, Jean G. Previnaire, Alpesh Patel, Giorgio Scivoletto, Lisa N. Sharwood, Andrea Townson, Susan Urquhart, Aki Vainionpää, Atiq Uz Zaman, Vanessa K. Noonan, Christiana L. Cheng

**Affiliations:** 1 Department of Neurological Surgery, Keck School of Medicine, University of Southern California, Los Angeles, CA USA; 2 Department for Spinal Cord Injuries, Rigshospitalet, University of Copenhagen, Copenhagen, Denmark; 3 Indian Spinal Injuries Centre, New Delhi, India; 4 Medical School University of São Paulo, Sao Paulo, Brazil; 5 Orthopedic Surgery Department, Hamad General Hospital, Doha, Qatar; 6 Department of Neurology and Rehabilitation, Tampere University Hospital, Tampere, Finland; 7 Department of Orthopaedics & Traumatology, Faculty of Medicine, University of Hong Kong, Hong Kong, SAR China; 8 Department of Rehabilitation Medicine, Peking University Third Hospital, Beijing, China; 9 John Walsh Centre for Rehabilitation Research, Faculty of Medicine and Health, University of Sydney, Sydney, NSW Australia; 10 Post-acute and Palliative Care Department Novi Marof, General Hospital Varaždin, Varaždin, Croatia; 11 Sina Trauma and Surgery Research Center, Tehran University of Medical Sciences, Tehran, Iran; 12 Physical Medicine & Rehabilitation, Dalhousie University Faculty of Medicine, Fredericton, NB Canada; 13 Fondation Hopale, Berck sur Mer, France; 14 Middlemore Hospital, Auckland, New Zealand; 15 Spinal Unit and Spinal Rehabilitation (SpiRe) Lab, IRCCS Fondazione S. Lucia, Rome, Italy; 16 University of Sydney, Sydney, NSW Australia; 17 Division of Physical Medicine and Rehabilitation, Department of Medicine, University of British Columbia, Vancouver, BC Canada; 18 Spinal Injuries Unit, Queensland Spinal Cord Injuries Services, Brisbane, QLD Australia; 19 Department of Medical Rehabilitation, Oulu University Hospital, Oulu, Finland; 20 Lahore Medical and Dental College, Ghurki Trust Teaching Hospital, Lahore, Pakistan; 21 Praxis Spinal Cord Institute, Vancouver, BC Canada

**Keywords:** Traumatic spinal cord injury, Healthcare services, International survey, High-income countries, Low- and middle-income countries, Indicators

## Abstract

**Background:**

To describe the key findings and lessons learned from an international pilot study that surveyed spinal cord injury programs in acute and rehabilitation facilities to understand the status of spinal cord injury care.

**Methods:**

An online survey with two questionnaires, a 74-item for acute care and a 51-item for rehabilitation, was used. A subset of survey items relevant to the themes of specialized care, timeliness, patient-centeredness, and evidence-based care were operationalized as structure or process indicators. Percentages of facilities reporting the structure or process to be present, and percentages of indicators met by each facility were calculated and reported separately for facilities from high-income countries (HIC) and from low and middle-income countries (LMIC) to identify “hard to meet” indicators defined as those met by less than two-thirds of facilities and to describe performance level.

**Results:**

A total of 26 acute and 26 rehabilitation facilities from 25 countries participated in the study. The comparison of the facilities based on the country income level revealed three general observations: 1) some indicators were met equally well by both HIC and LMIC, such as 24-hour access to CT scanners in acute care and out-patient services at rehabilitation facilities; 2) some indicators were hard to meet for LMIC but not for HIC, such as having a multidisciplinary team for both acute and rehabilitation settings; and 3) some indicators were hard to meet by both HIC and LMIC, including having peer counselling programs. Variability was also observed for the same indicator between acute and rehabilitation facilities, and a wide range in the total number of indicators met among HIC facilities (acute 59–100%; rehabilitation 36–100%) and among LMIC facilities (acute: 41–82%; rehabilitation: 36–93%) was reported.

**Conclusions:**

Results from this international pilot study found that the participating acute and rehabilitation facilities on average adhered to 74% of the selected indicators, suggesting that the structure and processes to provide ideal traumatic spinal cord injury care were broadly available. Recruiting a representative sample of SCI facilities and incorporating regional attributes in future surveys will be helpful to examine factors affecting adherence to indicators.

**Supplementary Information:**

The online version contains supplementary material available at 10.1186/s12913-022-08847-w.

## Background

Sustaining a traumatic spinal cord injury (tSCI) is a devastating neurological event resulting in physical impairments, functional limitations, psychosocial distress, and secondary complications affecting multiple body systems [1]. Access to acute care and rehabilitation that is specialized, timely, and patient-centered [2] is essential to minimize secondary injury, maximize recovery, and optimize community participation. The provision of such care requires coordinated efforts at all levels **-** health system, healthcare organizations, and programs (e.g., SCI acute/rehabilitation program) – with structures and processes in place to deliver the right care at the right time in the right place. This can be complex and challenging, not only for low- and middle-income countries (LMIC) but also high-income countries (HIC). As such, global efforts led by the World Health Organisation (WHO) and the International Spinal Cord Society (ISCoS) aim to promote health system strengthening through provision of guidance and resources [3] for countries to improve their response to tSCI [4].

In 2013, the WHO-ISCoS International Perspectives on Spinal Cord Injury (IPSCI) report was published [4], outlining a comprehensive range of needs in healthcare, social services, and policies for persons living with SCI around the world. This initiative provided evidence-based recommendations to address these needs at each of the system, organization, and program levels considering countries’ economic status. In 2017, the WHO launched the “Rehabilitation 2030″ initiative to support countries around the world in strengthening their health systems by increasing the quality and expand the accessibility of rehabilitation services [5]. In parallel with this global effort, ISCoS and WHO are collaborating to develop a SCI-specific Service Module as a practical toolkit for national health systems to complement the generic WHO Rehabilitation Guide for Action [3]. This toolkit will provide resources that can be used to guide the country with an assessment of the needs and opportunities (referred to a situation assessment) and to support development of a “National Roadmap” for SCI services strengthening and capacity-building. The Module will align with and refer to several WHO products, such as WHO’s Package of Interventions for Rehabilitation (PIR) for SCI [6] and Rehabilitation Competency Framework for workforce development in SCI [7].

Other initiatives are also underway to help plan and adapt implementation strategies to the local context, using a dynamic model where feedback informs health system improvements. The Learning Health System-International SCI Survey (LHS-InSCI) project is mapping the health system response to SCI in participating countries, as well as documenting the lived experience of persons with SCI to inform the IPSCI implementation plan [8]. The International Classification of Service Organizations in Rehabilitation (ICSO-R) is a framework for mapping rehabilitation services at the organization level to enable the description of available service organizations in a uniform way and facilitate the identification of rehabilitation services provision worldwide [9].

Missing from the current framework and implementation plan is an audit of the structures (e.g., equipment) and processes of care (e.g., protocols and adherence to protocols) that support the provision of healthcare services at the program level. To date, quantification of the structures and processes of SCI programs has been surveyed only within specific countries such as Canada [10], with limited studies comparing among countries [11]. These two studies documented differences in the degree of specialization of SCI care [10] and the availability of SCI speciality services [11]. Having a standardized way to quantify variations in structures and processes related to specialized SCI care on a global scale would provide insight as to the level of readiness and capacity of a SCI program to implement guidelines and recommendations, such as those in development by the WHO. This would assist in elucidating the organizational practices and existing variations, as well as overall barriers and drivers of practice change implementation. Further, a report on quantitative evaluation could identify high-performing facilities where learnings can be shared and baselines established to assist future improvement efforts.

The objective of this paper is to describe the key findings and lessons learned from an international pilot study that surveyed SCI programs in acute and rehabilitation facilities to understand the status of SCI care. Lessons learned and the international network established from this pilot study can inform ongoing SCI care global initiatives.

## Methods

### Study design

This pilot study utilized an environmental scan and solicited feedback via a survey from participating facilities. A published questionnaire on the structure and process attributes involved in the delivery of specialized care for tSCI in Canada [10] was adapted for this study through consultation with SCI experts involved in the project, including a neurosurgeon, clinical nurse specialist, physical therapist, and researcher. The adapted survey encompassed two questionnaires in English, a 74-item for acute care and a 51-item for rehabilitation. Response options included providing a numerical estimate or describing the service in a text field (Additional file 1). The questionnaire themes included specialized care (e.g., SCI expertise, multidisciplinary team), timeliness (e.g., timing of treatment, transition between the phases of care), patient-centered approach (e.g., mental health, peer support, and follow-up services), and the capacity to advance evidence-based care (e.g., access to data, guidelines). A survey user guide was created to provide additional information on certain questions.

Ethics approval was obtained from Veritas Independent Review Board and local institutional boards for participating facilities.

### Study sample and recruitment

Clinicians affiliated with an acute or rehabilitation facility treating tSCI were eligible to participate. Participating rehabilitation facilities were rehabilitation hospitals providing in-patient and out-patient rehabilitation services; there were no independent clinics (e.g., PT clinics) included. Given the environmental scan approach of this study, the only eligibility criterion was that the facility had to treat patients with SCI in the year of participation. Due to the lack of any comprehensive list or database of SCI hospitals/facilities in the world, a convenience sample was recruited using a snowball approach, with two consecutive rounds. First, international contacts known by Praxis Spinal Cord Institute, a Canadian-based not-for-profit organization, were invited. Subsequently, participants of the first round were asked to invite their colleagues and professional networks, including ISCoS, the African Spinal Cord Injury Network, the Asian Spinal Cord Network, the Latin American Network, and the National SCI Registry of Iran. Participation was voluntary and participants were offered a small honorarium to compensate them for the time required to complete the questionnaires.

### Data collection

The survey was securely administered online using the Global Research Platform [12] developed and supported by Praxis. Data was collected for the calendar year of enrolment (2013 or 2014) using hospital administrative data when available (e.g., Discharge Abstract Database in Canada), or best judgement from the participant (clinician’s experience) when no data was available. Upon submission of survey responses, data validation was performed by study investigators to resolve any unanswered questions and potential misinterpretations by follow-up inquiry. Submitted surveys were considered complete when all the mandatory questions were answered. Although the study started in 2014–2015, there were some challenges in recruitment and so the survey remained open until 2018 to ensure enrollment of facilities from underrepresented regions and continents.

### Indicators

After data collection, a descriptive summary of the survey data was presented to the participants to ensure it reflected tSCI care (i.e. face validity). Given the large amount of data due to more participants than anticipated, the coauthors made a post-hoc decision to select a subset of the survey items that aligned to the topics focused in the then-recently published International Perspectives on Spinal Cord Injury (IPSCI) [13], particulary on the themes of specialized care, timeliness, patient-centeredness, and evidence-based care. These items were operationalized as structure or process indicators (Tables 2 and 3) [14].

### Analysis

Characteristics of the participating facilities, including country income level, the region the facility serves in the country, funding source, hospital/facility size (number of beds), and patient volume were reported using frequencies and percentages for categorical variables, and medians and interquartile ranges (IQR) for continuous variables. The country income status was designated as HIC or LMIC, based on the gross national income per capita in calendar year 2013 reported by the World Bank [15]. Adherence to the indicators was reported as percentages of facilities that reported the structure or process to be present. “Hard to meet” indicators were defined as those met by less than two-thirds of facilities [16]. Percentages of indicators met by each facility were also calculated to assess performance level.

## Results

### Characteristics of participating facilities

A total of 16 acute care-only facilities, 10 facilities providing both acute and rehabilitation care, and another 16 rehabilitation-only facilities from 25 countries participated in the study, resulting in 26 responses for each of the acute care and rehabilitation surveys (Table 1). The majority of participating facilities were from HIC (60%). Most of the acute care facilities were part of Level I trauma centers, while two facilities from LMIC were part of Level II trauma centers. The majority of the rehabilitation facilities were part of a larger rehabilitation center offering multiple programs, while five facilities specialized in SCI only.

**Table 1:** Characteristics of participating facilities

	Acute ***N*** = 26	Rehab ***N*** = 26
Continent and region; %		
Africa		
Eastern Africa	4	4
Northern Africa	4	0
Southern Africa	4	4
Asia		
East Asia	8	8
Middle East	23	12
South Asia	12	8
Europe		
Eastern Europe	4	4
Northern Europe	12	12
Southern Europe	0	19
Western Europe	0	8
Oceania	19	8
North America	8	12
South America	4	4
Country income level; %		
High-income	50	73
Low- and middle-income	50	27
Geographic coverage; %		
Region-wide	23	19
Province/state-wide	31	35
Country-wide	46	46
Funding source; %		
Government only	46	38
Government and other	42	54
Non-government	12	8
Size of facility based on number of beds; median (IQR)		
Total number of beds in acute facility	592 (471)	N/A
Number of SCI beds in rehabilitation facility	N/A	33 (30)
Annual new tSCI admissions; median (IQR)	57 (80)	40 (36)
Unknown; %	4	N/A

*IQR* Interquartile range, *SCI* Spinal cord injury, *tSCI* Traumatic SCI

### Adherence to indicators

The adherence of indicators reported by acute care and rehabilitation facilities, stratified by country income level, are outlined in Tables 2 and 3, respectively. The comparison of the facilities based on the country income level revealed three general observations: 1) some indicators were met equally well by both HIC and LMIC, such as 24-hour access to CT scanners and operating rooms in acute care and out-patient services at rehabilitation facilities; 2) some indicators were hard to meet for LMIC but not for HIC, such as having a multidisciplinary team or standardized practice of care based on best available evidence (e.g., accreditation, protocols or guidelines) in both acute and rehabilitation settings; and 3) some indicators were hard to meet by both HIC and LMIC, including having peer counselling programs, regular use of data to inform SCI care, and housing accommodation by rehabilitation facilities while patients await home renovation.

**Table 2:** Percent of acute facilities by country income level meeting each indicator

Indicators	HIC ***N*** = 13	LMIC ***N*** = 13
Specialized care		
Classify tSCI with neurological examination (p)	77	*62*
Have a team of clinical staff with SCI expertise (s)	85	*46*
Have a multidisciplinary team (s)	85	*38*
Timeliness & transition		
Have a triage protocol for direct admission (p)	69	*31*
Have a direct relationship between acute SCI unit and referring rehab centers (s)	85	69
Have rehab facilities in acute unit (s)	92	*54*
Have spine surgeon on-call 24 h (s)	92	85
Have 24 h access to MRI (s)	100	*62*
Provide SCI medical follow-up (p)	100	92
Have 24 h access to CT scanner (s)	100	100
Have 24 h access to operating room (s)	100	100
Provide early rehab in acute setting (p)	100	100
Patient-centeredness		
Have a peer counsellor (s)	*54*	*23*
Have a psychologist (s)	85	*62*
Provide services for detection and treatment for mental health (p)	100	*62*
Capacity to advance evidence-based care		
Make regular use of data to inform SCI care (p)	*54*	*46*
Practice of care standardized to recommendations/guidelines (p)	69	*38*

Structure and process indicators are represented by (s) and (p), respectively. Italic represents “hard to meet” indicators. *HIC* High-income countries, *LMIC* Low- and middle-income countries, *SCI* Spinal cord injury, *tSCI* Traumatic SCI. All the indicators, except for those listed below, were a yes/no question

For “classifying tSCI with neurological examination”, it is considered as “yes” if responded using International Standards for Neurological Classification of SCI examination [17] or clinical definition (e.g., sensory-motor deficit)

For “have a multidisciplinary team”, it is considered as “yes” if indicated having all the following positions caring for patients with tSCI in acute: surgeon (spine, ortho- or neuro-), nurse (registered nurse, registered practice nurse, or licensed practice nurse), rehabilitation physician/ physiatrist, physiotherapist (PT), occupational therapist (OT), social worker or case manager, psychologist [18]; in rehabilitation: rehabilitation physician/physiatrist, PT, OT, nurses (registered nurse or licensed practice nurse), psychologist, speech-language pathologist, case manager/social worker [19]

For “provide services for detection and treatment for mental health”, it is considered as “yes” if indicated any of the following: monitor mental health/emotional wellbeing, provide education, screening with no assessment tool, screening with the use of standardized assessment tool, interview and diagnosis conducted by appropriate healthcare provider, intervention strategies, including medication, counselling/psychotherapy, exercise/activation, self-management, reassessment prior to discharge, train staff to recognize symptoms of depression, anxiety, post-traumatic stress disorder, etc

For “provide SCI medical/rehabilitation follow-up”, it is considered as “yes” if answered “regularly” or “as needed” to the question “do you provide SCI medical/rehabilitation-related follow-up services after discharge?”

**Table 3:** Percent of rehabilitation facilities by country income level meeting each indicator

Indicators	HIC ***N*** = 19	LMIC ***N*** = 7
Specialized care		
Have a multidisciplinary team (s)	74	*43*
Have a team of clinical staff with SCI expertise (s)	89	86
Timeliness & transition		
Have facilities for patients to wait for home renovation (s)	*37*	*29*
Provide SCI medical follow-up (p)	79	86
Provide SCI rehabilitation follow-up (p)	95	71
Have a direct relationship between rehab program and acute facilities (p)	100	86
Patient-centeredness		
Have a peer counsellor (s)	*58*	*43*
Provide out-patient rehabilitation (p)	89	86
Provide services for detection and treatment for mental health (p)	95	100
Have a psychologist (s)	100	71
Capacity to advance evidence-based care		
Make regular use of data to inform SCI care (p)	*58*	*43*
Practice of care standardized to recommendations/guidelines (p)	79	*43*
Collect data on service delivery (p)	84	71

Structure and process indicators are represented by (s) and (p), respectively. Italic represents “hard to meet” indicators. *HIC* High-income countries, *LMIC* Low- and middle-income countries, *SCI* spinal cord injury

For other indicators, the adherence varied remarkably between acute and rehabilitation facilities, implying differences in the level of readiness or capacity for adopting those indicators at the program level. In LMIC facilities, for instance, more rehabilitation facilities than acute facilities reported having a team of clinical staff with SCI expertise [18] (86% vs 46%) or providing mental health services (100% vs 62%) (Table 4).

**Table 4:** Percent of indicators met by facilities stratified by country income level

Mean (range)	All ***N*** = 26	HIC Acute ***N*** = 13; Rehab ***N*** = 19	LMIC Acute ***N*** = 13; Rehab ***N*** = 7
Acute facilities	74% (41–100%)	85% (59–100%)	63% (41–82%)
Rehabilitation facilities	74% (36–100%)	78% (36–100%)	64% (36–93%)

*HIC* High-income countries, *LMIC* Low- and middle-income countries

**Fig. 1:**
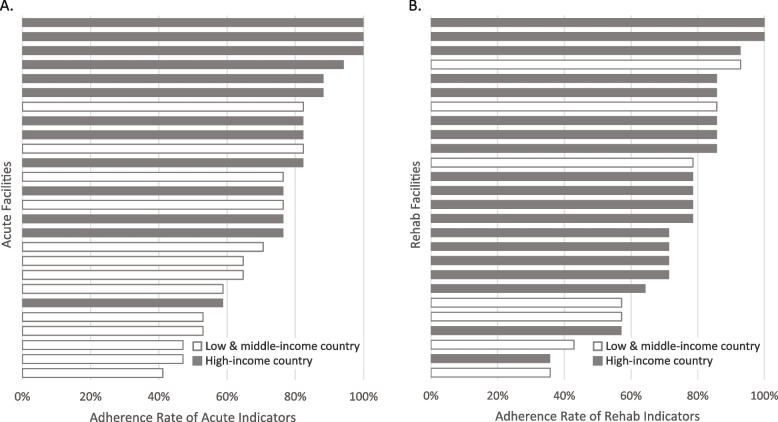
Percent of indicators met by each participating facility by country income level (A. acute facilities, *n* = 13 in high-income country (HIC), *n* = 13 in low and middle-income country (LMIC); B. rehabilitation facilities, *n* = 19 in HIC, *n* = 7 in LMIC)

There were also differences in the total number of indicators met by facilities. In HIC, three of 13 acute care facilities reported meeting all indicators, while one met only 59% of indicators (10 of 17) (Fig. 1A). Among the 13 acute care facilities in LMIC, the majority met fewer than 60% of indicators, although there were two high-performing facilities that met 82% of indicators (14 of 17), and there were three that performed similarly as seen in HIC. Among the 19 rehabilitation facilities located in HIC, two met all the rehabilitation indicators, while one met only 36% of indicators (5 of 14), which was also the lowest adherence reported from LMIC (Fig. 1B).

## Discussion

Timely delivery of specialized care for tSCI has been proven to improve the neurological outcomes and decrease the burden on healthcare systems and societies [18]. Yet, studies of healthcare access, related standards, and quality attributes of care provided for individuals with tSCI are scarce and mostly limited to HIC. This survey examined tSCI care, specifically structure and process attributes of SCI programs, in facilities across six continents and included countries with different levels of resources and economic development. Despite the various challenges that were encountered, this pilot study demonstrated feasibility of engaging Heads or Directors of SCI programs or their delegates and collecting data on care across the globe, covering various aspects of SCI services and processes along the continuum of care. The structure and processes of care (indicators) could be further refined to operationalize the current IPSCI recommendations for measurement and comparison. This study provided some insights that might inform the implementation of future global initiatives aimed at measurement and improvement of SCI care.

### Adherence to indicators

We demonstrated that both acute care and rehabilitation facilities in this study on average adhered to 74% of the selected indicators, implying that the structure and processes to provide specialized, timely, patient-centered, and evidence-based care were broadly available in the majority of participating facilities. Although the LMIC facilities in this study do not represent most LMIC facilities given our sampling bias as discussed in lessons learned below, the similarities between the LMIC and HIC facilities in terms of their level of adherence to some of the indicators support the notion that abundance of resources may not necessarily equate to unrestricted access, as gaps in coverage and accessibility may pose barriers to timeliness and quality of SCI care [20]. Nevertheless, economic limitations result in healthcare disparity, while priorities in resource allocation vary widely across healthcare systems, due to fundamental differences in social, cultural, and financial factors. Inherent to these differences, the acceptable level of care quality also differs between countries [21].

### Regional context

In the attempt to identify underlying factors that might affect the adherence to indicators, we asked about the characteristics of the participating facilities including geographic coverage, number of beds, and hospital/facility funding sources. However, there are additional regional attributes that provide context for interpretation of the data, such as the regional incidence of tSCI and the health system funding model. Traditional or cultural variations in training, workforce, and practice might also impact care, such as the team composition and role delineation by profession. Due to the paucity of literature (e.g., tSCI incidence, professional variations) and the complexity (e.g., funding model) of these regional attributes, their impact on adherence to indicators is challenging to assess and require further research. Working with regional societies and leveraging the ongoing efforts of LHS-InSCI initiative to integrate perspectives of the countries health system and individuals with SCI will enhance the relevance of the survey questions and indicators.

### Survey structure

The requirement to complete the survey in English limited broader recruitment of certain countries from South America and Asia, further biasing our sample. Translation of the survey would increase participation in these countries. During the survey rollout, interpretive variations among the participants were encountered in areas such as taxonomy (e.g., definition of direct admission), professional role designations (e.g., for physiatrists), and data sources (e.g., clinical judgement vs. administrative data). In retrospect, involving experts in different aspect of SCI care (e.g., physiatry), decision makers, persons with lived experience, as well as experts in survey design, and testing of the survey in a subset of countries across different regions would have been a key early step to ensure clarity of the survey terminology and consistency in definitions. Compared to the electronic dissemination of the study protocol at the beginning of this study, having orientation meetings would have been helpful to address any inconsistencies and engaging participants throughout the study would have facilitated data collection.

It is important to balance the level of detail in the survey with the feasibility of completing it [22], since the inclusion of nice-to-know components is likely to pose additional burden on the participants and complicate the data analysis. Our survey covered a wide range of SCI care attributes, such as admission or discharge process, care services, and staffing. While a multi-faceted approach is required to understand whether a certain recommendation can be met, parts of our survey may have lacked sufficient granularity. For instance, in terms of timely decompression, having 24-hour access to the operating room and surgeon would not be conducive to timely decompression without knowing the availability status of the surgical staff. Similarly, having a triage protocol in place is not informative without knowing the rigor in implementation of such protocols. Furthermore, outcome indicators (e.g., time to admission or decompression) are also needed to ascertain if recommendations are met. Accordingly, prioritization of the research questions within the survey would assist with structuring the questions (e.g., free text vs multiple choice) and determining a core set of structure, process and outcome indicators for a streamlined and thorough survey.

### Lessons learned

To describe the current state of SCI care, it is important to have a representative sample of care facilities. In our experience, connecting with clinicians at the acute and rehabilitation centers was helpful to identify individuals who were best to complete the various sections of the survey. Despite our efforts, inclusion of an unbiased sample was challenging due to the limited representativeness and generalizability in the members of our network. This issue may be due to the inclusion of mostly urban Level I trauma centers in this study. Therefore, efforts are needed to connect to private facilities as well as those located in LMIC or rural regions, especially in healthcare systems which do not have centralized care for individuals with SCI.

Based on our experience, individuals with an academic appointment with research interest were more likely to participate in the survey. This pattern may have led to responder bias due to the higher recruitment of facilities with extensive resources and special interest in the area in both HIC and LMIC regions. It could be anticipated that the facilities not captured in this study may have met fewer indicators, not have the capacity to collect data or access to the data required, or were limited by language barriers. The study recruitment timeframe was extended to increase the sample size at the risk of the collected data becoming outdated; although significant changes in practice are presumably unlikely to occur over the short term. In future, a sampling framework and a recruitment plan should be developed prior to survey roll-out to ensure a representative sample. An additional step for international initiatives involving LMIC is to consider the digital competency and capacity (e.g., internet access) of the potential participants, and accommodating their needs especially in rural areas. Our survey offered an honorarium to compensate the participant for the time taken collecting data. The honorarium could have served as an incentive for some participants working in more financially constrained facilities, acting as a possible source of bias. For example, one of our facilities reported leveraging the funds to build a shed for wheelchair storage. However, it is unclear whether this assisted recruitment.

### Recommendations

Building a strong collaboration with WHO/ISCoS and other international organizations is crucial for future global initiatives on measuring SCI care. ISCoS supports regional networks through workshops and symposia to share knowledge and support SCI care [23]. Thus, leveraging their extensive networks will likely enhance the recruitment and participation in future studies. This approach will ensure smaller and rural hospitals/facilities that treat SCI (e.g., through the International Classification of Diseases codes) are included in addition to the urban Level I/II trauma centers to ensure representation across countries and regions.

Information on the structure and processes of care does not provide any information on the impact on outcomes. Future work should include development of comprehensive quality assessment frameworks and engaging international stakeholders (e.g., the WHO-ISCoS SCI Service Module Advisory Group), policy makers, caregivers, and persons living with SCI. Such efforts should be centered around actionable indicators for care practices that can be relatively easy to optimize and impact outcomes, while omitting indicators that are hard to collect, as well as those already scoring well by most countries, some of which were highlighted in this study.

In order to translate research findings to quality improvement efforts, it is crucial to have evidenced-based indicators and identify the various factors that influence the adherence to guidelines (e.g., traumatic brain injury (TBI) [24]). In TBI, a survey on structure and processes of care among centers in the CENTER-TBI study revealed substantial variation in care [25], on which the development of a set of quality indicators was based, together with international guidelines and clinical expertise [24]. Testing the indicator set in over fifty intensive care units for its potential for quality measurement and improvement suggested that the indicators with high between-center variation and suboptimal adherence rates could be useful for benchmarking and improving quality of care [26]. The empirical evidence supporting the successful implementation of quality improvement initiatives in TBI [26] corroborates that a similar approach is sustainable for SCI. In this regard, surveying facilities at regular intervals would quantify the progress (e.g., improvement of practice and adoption of guidelines) and enable economic evaluations. Inclusion of patient outcomes, such as patient-reported outcome measures and clinical outcomes aligned to the International SCI Data Sets [27] would also help understand if indicators are linked to improved services and capture the impact of guidelines on patients’ health and quality of life.

Rapid audit and feedback cycles can be incorporated in future studies as an incentive and motivation to participate. A few key indicators can be selected to monitor internationally and used to generate “report cards” for facilities to prioritize areas of improvement and advocate for resources. Future quality assessment surveys should be viewed as an opportunity to profile high-performing facilities, especially those located in LMIC, to better understand the facilitators and motivate other facilities with similar characteristics. In our study, some of the LMIC facilities successfully met as many indicators as their HIC counterparts.

### Future directions

As demonstrated by our pilot study, the evaluation of the structure and processes of SCI care is feasible. However, this study highlighted that it would be important to engage ISCoS and WHO (e.g., through the SCI Service Module) and collaboration with ongoing international initiatives (e.g., LHS-InSCI) to ensure a representative sample of SCI facilities and an efficient process. The international network built because of this study was an unintended yet significant achievement demonstrating the strong desire for international collaboration that could benefit future initiatives. Development of a unified strategy to implement a ‘living’ benchmarking survey that incorporates SCI services internationally and allows for comparisons, harmonization, and improvement of care could be realized through such collaborations. With regular reporting and feedback loops, this approach would assist with the monitoring and evaluation of implementation efforts to improve health system response in SCI care worldwide.

## Conclusions

Results from this international pilot study found that the participating acute and rehabilitation facilities on average adhered to 74% of the selected indicators, suggesting that the structure and processes to provide ideal tSCI care were broadly available. Recruiting a representative sample of SCI facilities and incorporating regional attributes in future surveys will be helpful to examine factors affecting adherence to indicators. Despite the wide availability of evidence-based practice guidelines for tSCI, a facility-level quality assessment framework with objective and actionable indicators has yet to be developed. Lessons learned and the international network established from this pilot study can inform ongoing SCI care global initiatives.

## Supplementary Information

**Additional file 1.** Survey questionnaire of spinal cord injury (SCI) care in acute and rehabilitation facilities in the study.

## Data Availability

The datasets used and/or analyzed during the current study are not publicly available due to limitations of ethical approval involving anonymity of participating facilities but are available from the corresponding author on reasonable request.

## Abbreviations

HIC: High-income countries
ICSO-R: International Classification of Service Organizations in Rehabilitation
IPSCI: International Perspectives on Spinal Cord Injury
IQR: Interquartile range
ISCoS: International Spinal Cord Society
LHS-InSCI: Learning Health System-International Spinal Cord Injury Survey
LMIC: Low- and middle-income countries
OT: Occupational therapist
PIR: WHO’s Package of Intervention for Rehabilitation
PT: Physiotherapist
SCI: Spinal cord injury
TBI: Traumatic brain injury
tSCI: Traumatic spinal cord injury
WHO: World Health Organization
