# Supplementary material for: An international survey of the structure and process of care for traumatic spinal cord injury in acute and rehabilitation facilities: lessons learned from a pilot study
Source: BMC Health Serv Res. 2022 Dec 21;22:1565. doi: 10.1186/s12913-022-08847-w (PMC9768992; doi:10.1186/s12913-022-08847-w)
Supplement: Supplementary file 1 — Additional file 1. Survey questionnaire of spinal cord injury (SCI) care in acute and rehabilitation facilities in the study. [file 12913_2022_8847_MOESM1_ESM.pdf]

An International Survey of the Structure and Process of Care for Traumatic Spinal Cord Injury in Acute and Rehabilitation Facilities: Lessons Learned from a Pilot Study (Abedi *et al.*)

Additional File 1. Survey questionnaire of spinal cord injury (SCI) care in acute and rehabilitation facilities in the study.

Survey Questionnaire of Spinal Cord Injury Care in Acute Facilities	
**Question**	**Answer**
**ACUTE CARE – ADMINISTRATIVE INFORMATION**	
1. Name of hospital	
2. Address	
3. Health authority	
4. Trauma level of hospital<br>Definition of Trauma Levels (American Trauma Society):<br>• Level I – a tertiary care facility central to the trauma system<br>• Level II – a centre that is able to initiate definitive care for all injured patients<br>• Level III/IV – a centre that has demonstrated an ability to provide advanced trauma life support prior to transfer of patients to a higher level trauma centre	Select one that applies:<br>• Level I<br>• Level II<br>• Level III/IV
5. Region served	Select one that applies:<br>• Region-wide<br>• Province/state-wide<br>• Country-wide
6. Total number of beds in the hospital	
7. Number of beds available for traumatic SCI admissions	
8. Please describe:<br>A) any important clinical or administrative changes relevant for traumatic SCI admission that may affect the data or answers in this survey and,<br>B) reasons for these changes	A)<br>B)
9. Annual number of traumatic SCI admissions	
10. Do you also care for non-traumatic SCI?	Yes/No
11. A) How do you classify traumatic SCI?	Select all that apply:<br>• ICD-9 or ICD-10 (WHO)<br>• Clinical definition (specify)<br>• Other (specify)
B) How do you classify non-traumatic SCI?	Select all that apply:<br>• ICD-9 or ICD-10 (WHO)<br>• Clinical definition (specify)<br>• Other (specify)
12. Number of direct traumatic SCI admission in a year (i.e. those arriving directly from scene of injury)	

Survey Questionnaire of Spinal Cord Injury Care in Acute Facilities	
**Question**	**Answer**
13. Number of non-direct traumatic SCI admission in a year (i.e. those arriving via peripheral/community hospital)	
14. For non-direct admissions, list the top 5 referring hospitals, in decreasing order of patient admissions.	
15. Upon admission, what is the first department/ward patients can be admitted to?<br>A) Patients arriving directly from scene of injury<br>B) Patients arriving via peripheral/community hospital	A)
	B)
**ACUTE CARE – ADMISSION OF PATIENTS TO A SPECIALIZED CENTRE**	
16. Is there a protocol in place to send patients with traumatic SCI, if identified at scene, directly to a specialized SCI centre?<br>Provide comments if needed.	Yes/No<br>Comments:
17. A) Are patients with traumatic SCI, if identified at scene, transported directly to a specialized SCI centre?<br>B) If no, why not?	A) Yes/No
	B)
18. What are the biggest challenges in getting patients to a specialized acute spinal unit in a timely fashion?	
19. How many patients with traumatic SCI, if any, did not meet admission criteria and were sent to another hospital for acute care?	
20. Please describe common reasons for not meeting admission criteria.	
**ACUTE CARE – TIMING OF TREATMENT**	
21. Does your centre endeavor to decompress (closed or open) cervical SCI within 24h of injury for patients with the following neurological scenarios?<br>(AIS – American Spinal Injury Association Impairment Scale)	A) For AIS A: Yes/No/Case-specific
	B) For AIS B – D: Yes/No/Case-specific
	C) For Central Cord Syndrome: Yes/No/Case-specific
22. Does your center endeavor to decompress (closed or open) thoracolumbar SCI within 24h of injury for patients with the following neurological scenarios?	A) For AIS A: Yes/No
	B) For AIS B – D: Yes/No
23. Does your center have 24h access to MRI (magnetic resonance imaging)?	Yes/No
24. Does your center have 24h access to operating room?	Yes/No
25. Is a spine surgeon on-call 24h?	Yes/No
**ACUTE CARE – FUNDING**	
26. Do patients have universal access to receive care at trauma/rehab/specialized centres?	Yes/No
27. What is the estimated percentage of admitted patients with SCI who are privately insured?	
28. What is the estimated percentage of admitted patients with SCI who are publicly insured?	

Survey Questionnaire of Spinal Cord Injury Care in Acute Facilities	
**Question**	**Answer**
29. What are the funding sources for the hospital?	Select all that apply:<br>• Government<br>• Government compensation<br>• Private insurance compensation<br>• Self-funding<br>• Organizational-funding<br>• Other (please specify)
30. What is the approximate percentage of funding that goes to the SCI unit?	
**ACUTE CARE – SERVICE AVAILABILITY**	
31. A) Number of spine orthopaedic surgeons (fellowship trained)<br>B) Number of spine neurosurgeons (fellowship trained)<br>C) Number of spine fellows in training	A)<br>B)<br>C)
32. Total number of CT (computerized tomography) scanners in hospital	
33. Number of CT scanners located in the emergency department (ED)	
34. A) What are the hours of operation for CT scanners (in hospital, in ED) on Mon-Fri and on Sat-Sun?<br>B) Is it available on-call after hours?	
35. Total number of MRI scanners in hospital	
36. A) What are the hours of operation for MRI scanners (in hospital, in ED) on Mon-Fri and on Sat-Sun?<br>B) Is it available on-call after hours?	
37. What is the usual practice for patients with traumatic SCI getting a CT scan or MRI, both on admission and during their acute care stay? (Please include practice for patients who already have completed a scan at a previous hospital, policies for prioritizing patients, role of level of injury, pre/post operation, etc.)	
38. Number of Operating Rooms (OR)	
39. Number of OR dedicated to trauma	
40. Number of OR that can be used for spine surgery	
41. Number of OR dedicated to spine surgery	
42. What wards can patients with traumatic SCI go to during their hospital stay in order of the patient flow (including Intensive care unit (ICU) & step-down unit)?	

Survey Questionnaire of Spinal Cord Injury Care in Acute Facilities	
**Question**	**Answer**
43. For ICU and the wards mentioned above, please indicate:<br>A) Name of ward<br>B) Total number of beds<br>C) Beds available for traumatic SCI admissions<br>D) Nurse to patient ratio for SCI beds<br>E) Are all SCI beds staffed at the same ratio?<br>F) Are patients with traumatic SCI grouped together in this ward?<br>G) If yes, what is the rationale for grouping?<br>H) Are early rehab services provided in this ward?<br>I) If yes, please highlight the differences between the rehab services provided in this ward/unit and those provided in inpatient rehab centre.<br>J) Provide frequency of rehab services (# hours/day, days/week, Mon-Fri only or every day).	
44. A) Do you have a team of clinical staff with SCI expertise (e.g. ISNCSCI training, pressure ulcer management) that look after patients with SCI?<br>B) If yes, describe the type of SCI expertise.	A) Yes/No<br>B)
45. Describe clinical positions that look after patients with SCI. Select all that apply and enter %FTE (Full-time equivalent) or by consult:	

**Clinical Position**	**√**	**% FTE / by consult**	**Clinical Position**	**√**	**% FTE / by consult**
Spine surgeon			Physiotherapy assistant		
Orthopaedic surgeon			Occupational therapist		
Neurosurgeon			Occupational therapy technician		
Resident			Occupational therapy assistant		
Clinical nurse specialist			Speech language pathologist		
Advanced practice nurse			Social worker		
Clinical nurse educator			Dietitian/Nutritionist		
Registered nurse			Pharmacist		
Registered practice nurse			Respiratory therapist		
Licensed practice nurse			Respirologist		
Intensivist			Spiritual care		
Case manager			Psychologist		
Transition services			Neuropsychologist		
Patient care manager			Administrative assistant		
Patient care coordinator			Counsellor/Coordinator		
Physiatrist/Rehab physician			Peer counsellor		
Physiotherapist			Other (please specify)		

46. Describe the role of physiatrist/rehab physician in acute care. (i.e. Perform assessment regularly or by consult)	
47. Are there any rehab facilities (e.g. rehab gym) located in acute care setting?	Yes/No

Survey Questionnaire of Spinal Cord Injury Care in Acute Facilities	
**Question**	**Answer**
48. A) Is there a clinician/coordinator who manages the patient's entire care journey?	A) Yes/No
B) If yes, select all that apply:	B) Select all that apply:<br>• Surgeon<br>• Physiatrist/rehab physician<br>• Staff from SCI organization (coordinator, peer counsellor)<br>• Other (please specify)
49. Do you have services for detection and treatment for mental health?	Select all that apply:<br>• Monitor mental health/emotional wellbeing<br>• Provide education<br>• Screening with no assessment tool<br>• Screening with the use of standardized assessment tool<br>• Interview and diagnosis conducted by appropriate healthcare provider<br>• Intervention strategies including medication, counselling/psychotherapy, exercise/activation, self-management<br>• Reassessment prior to discharge<br>• Train staff to recognize symptoms of depression, anxiety, post-traumatic stress disorder, etc.<br>• Other (please specify)
50. A) Do you provide SCI medical-related follow-up services after discharge?	A) Select all that apply:<br>• Regularly<br>• As needed<br>• Limited<br>• No
B) If yes, how is the follow-up conducted?	B) Select all that apply:<br>• Telemedicine<br>• Consultation between healthcare practitioners and patient (at distant healthcare centre or at home)<br>• Assessment and monitoring of patient (at distant healthcare centre or at home)<br>• Therapy delivery to patient (at distant healthcare centre or at home)<br>• Other applications (please specify)

<table>
<tr><th colspan="2">Survey Questionnaire of Spinal Cord Injury Care in Acute Facilities</th></tr>
<tr><th>Question</th><th>Answer</th></tr>
<tr><td rowspan="2">51. A) Do you provide SCI rehab-related follow-up services after discharge?

B) If yes, how is the follow-up conducted?</td><td>A) Select all that apply:
• Regularly
• As needed
• Limited
• No</td></tr>
<tr><td>B) Select all that apply:
• Telemedicine
• Consultation between healthcare practitioners and patient (at distant healthcare centre or at home)
• Assessment and monitoring of patient (at distant healthcare centre or at home)
• Therapy delivery to patient (at distant healthcare centre or at home)
• Other applications (please specify)</td></tr>
<tr><td>52. What is the time-range of your follow-up services?</td><td>Select all that apply:
• < 1 year
• 1-3 years
• < 5 years
• < 10 years
• > 10 years
• Not applicable</td></tr>
<tr><th colspan="2">ACUTE CARE – RELATIONSHIP WITH REFERRING REHABILITATION CENTRE</th></tr>
<tr><td>53. List the top 3 intensive inpatient rehab centres where you refer your patients to, in decreasing order of referrals.</td><td></td></tr>
<tr><td>54. Is there a direct relationship between your acute SCI unit and the referring inpatient rehab centre(s)?</td><td>Select all that apply:
• Yes, with at least one referring centre
• Yes, with most-all referring centres
• No</td></tr>
<tr><td>55. If yes, is the relationship formal or informal?

Provide comments if needed.</td><td>Select all that apply:
• All formal (e.g. signed agreement)
• All informal
• Depending on the centre

Comments:</td></tr>
<tr><td>56. Please describe other attributes of the relationship, if any, between your acute SCI unit and the major referring inpatient rehab centre?</td><td>Select all that apply:
• Same building
• Same hospital centre
• Same city/region
• Same health authority
• Integrated clinical staff working closely together
• Same administrative staff</td></tr>
</table>

Survey Questionnaire of Spinal Cord Injury Care in Acute Facilities	
**Question**	**Answer**
57. How geographically close is your centre to the major referring rehab centre (in km)?	
58. Describe the processes for rehab referral.<br>A) Who requests the referral?<br>B) When is the request made?<br>C) What are the admission criteria for rehab?<br>D) After the referral has been requested, describe the subsequent steps leading up to rehab admission.	A)<br>B)<br>C)<br>D)
59. A) Were there any patients who were referred but did not meet the rehab admission criteria?<br>B) If yes, please list the main reasons in order of priority.	A) Yes/No<br>B)
60. Where did these patients go?	Select all that apply:<br>• Another rehab centre<br>• Long-term care centre<br>• Another ward within the hospital<br>• Another acute hospital<br>• Transitional centre<br>• Other (please specify)
61. A) Do these places provide rehab services?<br>B) If not, please comment on the process of delivering rehab services to these patients.	A) Yes/No<br>B)
62. A) Are there any gaps or barriers in ensuring these patients receive rehab services?<br>B) If yes, please describe common gaps or barriers.	A) Yes/No<br>B)
63. For patients who were accepted by the rehab centre, what was the number of patients put on waitlist and the min, max, and average wait time for rehab admission?	
64. A) Were patients transferred out of your centre temporarily to await rehab admission?<br>B) If yes, where were they sent to and what were the percentages?	A) Yes/No<br>B) Enter a percentage for each applicable selection:<br>• Another rehab centre<br>• Long-term care centre<br>• Acute hospital<br>• Transitional centre<br>• Other (please specify)
65. For patients who were transferred out of your centre temporarily to await rehab admission, were they readmitted to your centre before going to the rehab centre?	Yes/No/Sometimes
66. What are the common barriers to rehab admission?	

Survey Questionnaire of Spinal Cord Injury Care in Acute Facilities	
**Question**	**Answer**
**ACUTE CARE – DISCHARGE PROCESS**	
67. How many patients were discharged to the following destinations after acute care in a year?	Enter separately for patients with traumatic SCI and those with non-traumatic SCI:<br>• Home<br>• Inpatient rehab centre<br>• Another acute hospital<br>• Long-term care centre<br>• Assisted living centre<br>• Group living centre<br>• Transitional centre<br>• In-hospital death<br>• Other (please specify)
68. What are common barriers to discharge from acute care?	
**ACUTE CARE – STANDARDS OF CARE**	
69. A) Is the practice of care standardized in your country/region (e.g. accreditation, clinical practice guidelines/evidence-based protocols)?<br>B) If yes, provide brief details of standards available.<br>C) If yes, are the standards mandatory to follow?<br>D) If not, does your centre follow the standards?	A) Select one that applies:<br>• Yes<br>• No<br>• Not yet (in development) (specify)
	B)
	C) Yes/No
	D) Yes/No
**ACUTE CARE – ACCESS TO DATA**	
70. A) Do you submit data to any SCI registry?<br>B) If yes, provide name of database.	A) Select all that apply:<br>• National SCI database<br>• Regional SCI database<br>• Local SCI database<br>• Other (please specify)<br>• No, do not submit data
	B)
71. A) Do you have access to data/registry that can inform SCI care in your centre?<br>B) If yes, how often do you make use of the data?	A) Yes/No
	B) Select one that applies:<br>• Regularly<br>• Annually<br>• Rarely<br>• Never

Survey Questionnaire of Spinal Cord Injury Care in Acute Facilities	
**Question**	**Answer**
72. How is data on adverse events/complications experienced by patients collected?	Select one that applies:<br>• Recorded in notes but not collated or audited<br>• Recorded in notes and retrospectively audited<br>• Prospectively recorded in a database and audited<br>• Other (please specify)
**ACUTE CARE – CONCLUSION**	
73. What are some great challenges with regards to SCI care encountered by your centre?	
74. What are some efficiencies with regards to SCI care from your centre that can serve as a model for other centres?	

PRAXIS Spinal Cord Institute

Survey Questionnaire of Spinal Cord Injury Care in Rehabilitation Facilities	
**Question**	**Answer**
**REHAB CARE – ADMINISTRATIVE INFORMATION**	
1. Name of centre	
2. Address	
3. Health authority	
4. Region served	Select one that applies:<br>• Region-wide<br>• Province/state-wide<br>• Country-wide
5. Total number of beds in centre	
6. Number of beds available for SCI admissions	
7. Please describe:<br>A) any recent important clinical or administrative changes relevant to SCI admission that may affect the data or answers in this survey, and<br>B) reasons for these changes	A)<br>B)
8. What are the admission criteria?	
9. If applicable, how has the admission criteria changed over the years?	
10. Annual admissions of all patients	
11. Annual number of ***traumatic*** SCI admissions	
12. Annual number of ***non-traumatic*** SCI admissions	
13. How many patients with traumatic SCI, if any, did not meet rehab admission criteria?	
14. Please describe main reasons for not meeting admission criteria.	
15. A) Were there instances of patients with traumatic SCI having to wait for admission?<br>B) If yes, how many patients were on a wait list for rehab admission?<br>C) What was the min, max, and average wait time?	A) Yes/No<br>B)<br>C)
16. What were the main reasons for delay in order of priority?	
17. How do you prioritize patients on waitlist?	
18. A) Were any patients with traumatic SCI, if any, sent to another centre to await admission?<br>B) If yes, where were they sent to and what were the percentages?	A) Yes/No<br>B) For each of the selection, enter a percentage:<br>• Another rehab centre:<br>• Long-term care centre:<br>• Acute hospital:<br>• Transitional centre:<br>• Other (please specify):

Survey Questionnaire of Spinal Cord Injury Care in Rehabilitation Facilities	
**Question**	**Answer**
**REHAB CARE – RELATIONSHIP WITH ACUTE HOSPITAL**	
19. How many acute hospitals do you receive your patients with traumatic SCI from?	Select all that apply:<br>• 1<br>• <3<br>• <10<br>• >10
20. For all SCI admissions, list the top 5 referring hospitals, in decreasing order of patient admissions.	
21. Is there a direct relationship between your centre and the acute hospital(s)?	Select all that apply:<br>• Yes, with at least one referring hospital<br>• Yes, with most-all referring hospitals<br>• No
22. If yes, is the relationship formal or informal?	Select all that apply:<br>• All formal (e.g. signed agreement)<br>• All informal<br>• Depending on the hospital
23. Please describe other attributes of the relationship, if any, between your centre and the major SCI acute hospital?	Select all that apply:<br>• Same building<br>• Same hospital centre<br>• Same city/region<br>• Same health authority<br>• Integrated clinical staff working closely together<br>• Same administrative staff
24. How geographically close is your centre to the major SCI acute hospital (in km)?	
25. Please describe the referral process and the subsequent steps leading up to rehab admission.	
26. What are the common barriers to rehab admission?	
**REHAB CARE – FUNDING**	
27. Do patients have universal access to receive care in rehab/specialized centres?	Yes/No
28. What is the estimated percentage of admitted patients with SCI who are privately insured?	
29. What is the estimated percentage of admitted patients with SCI who are publicly insured?	
30. What are the funding sources for your centre?	Select all that apply:<br>• Government<br>• Government compensation<br>• Private insurance compensation<br>• Self-funding<br>• Organizational-funding<br>• Other (please specify)
31. What is the approximate percentage of funding that goes to the SCI unit?	

Survey Questionnaire of Spinal Cord Injury Care in Rehabilitation Facilities	
**Question**	**Answer**
**REHAB CARE – SPINAL UNIT STAFFING**	
32. Do you have a designated spinal unit defined as a physical space where patients with SCI are grouped together?	Yes/No
33. What physical areas/units/wards can patients with SCI be admitted to?	
34. What is the nurse to patient ratio in the wards described above?	
35. Are all SCI beds staffed at the same ratio on the wards described above?	
36. A) Do you have a team of clinical staff with SCI expertise (e.g. ISNCSCI training, pressure ulcer management) that look after patients with SCI?	A) Yes/No
B) If yes, describe the type of SCI expertise.	B)

37. Describe clinical positions that look after patients with SCI. Select all that apply and enter %FTE (Full-time equivalent) or by consult:

	√	% FTE / by consult		√	% FTE / by consult
Physiatrist/Rehab physician			Peer counsellor		
Primary care physician			Pharmacist		
Clinical nurse specialist			Speech language pathologist		
Advanced practice nurse			Neuropsychologist		
Clinical nurse educator			Psychiatrist		
Registered nurse			Psychologist		
Licensed practical nurse			Counsellor		
Occupational therapist			Urologist		
Physiotherapist			Internist		
Occupational therapy assistant			Geriatrician		
Physiotherapy assistant			Rehab engineer		
Therapeutic recreation therapist			Sexual health therapist		
Rehab therapist			Vocational therapist		
Respiratory therapist			Massage therapist		
Dietitian/Nutritionist			Research staff		
Social worker			Other (please specify)		
Case manager/Care management leader					

38. Describe the number of clinical positions on per shift:	• Physiatrist/Rehab physician<br>• Clinical nurse specialist<br>• Registered nurse<br>• Physiotherapist<br>• Occupational therapist

<table>
<tr><th colspan="2">Survey Questionnaire of Spinal Cord Injury Care in Rehabilitation Facilities</th></tr>
<tr><th>Question</th><th>Answer</th></tr>
<tr><th colspan="2">REHAB CARE – SERVICE AVAILABILITY</th></tr>
<tr><td>39. What rehab programs do you offer?</td><td>Select all that apply:
• Acquired brain injury
• Amputee
• Neuromusculoskeletal
• Neurotrauma
• Spinal cord injury
• Stroke
• Other (please specify)</td></tr>
<tr><td>40. What are the rehab facilities (e.g. rehab gym) located in your centre?</td><td></td></tr>
<tr><td>41. What are the typical hours of OT and PT therapy provided per week and the typical length of stay (LOS)?</td><td>Please describe separately for:
• Complete tetraplegia
• Incomplete tetraplegia
• Complete paraplegia
• Incomplete paraplegia</td></tr>
<tr><td rowspan="3">42. A) For patients who develop complications during rehab care, are they transferred back to the acute hospital?

B) If yes, are their beds held for the patients during service interruption?

C) How long are the beds held for?</td><td>A)</td></tr>
<tr><td>B)</td></tr>
<tr><td>C)</td></tr>
<tr><td>43. Do you have services for detection and treatment for mental health?</td><td>Select all that apply:
• Monitor mental health/emotional wellbeing
• Provide education
• Screening with no assessment tool
• Screening with the use of standardized assessment tool
• Interview and diagnosis conducted by appropriate healthcare provider
• Intervention strategies, including medication, counselling/psychotherapy, exercise/activation, self-management
• Reassessment prior to discharge
• Train staff to recognize symptoms of depression, anxiety, post-traumatic stress disorder, etc.
• Other (please specify)</td></tr>
<tr><td>44. A) Do you provide SCI medical-related follow-up services after discharge?</td><td>A) Select all that apply:
• Regularly
• As needed
• Limited
• No</td></tr>
</table>

Survey Questionnaire of Spinal Cord Injury Care in Rehabilitation Facilities

Question	Answer
B) If yes, how is the follow-up conducted?	B) Select all that apply:<br>• Telemedicine<br>• Consultation between healthcare practitioners and patient (at distant healthcare centre or at home)<br>• Assessment and monitoring of patient (at distant healthcare centre or at home)<br>• Therapy delivery to patient (at distant healthcare centre or at home)<br>• Other applications (please specify)
45. A) Do you provide SCI rehab-related follow-up services after discharge?<br><br>B) If yes, how is the follow-up conducted?	A) Select all that apply:<br>• Regularly<br>• As needed<br>• Limited<br>• No<br><br>B) Select all that apply:<br>• Telemedicine<br>• Consultation between healthcare practitioners and patient (at distant healthcare centre or at home)<br>• Assessment and monitoring of patient (at distant healthcare centre or at home)<br>• Therapy delivery to patient (at distant healthcare centre or at home)<br>• Other applications (please specify)
46. What is the time-range of the follow-up services?	Select all that apply:<br>• < 1 year<br>• 1-3 years<br>• < 5 years<br>• < 10 years<br>• > 10 years
47. Do you provide outpatient services for patients with SCI?<br><br>Provide comments.	Yes/No<br><br>Comments:

Survey Questionnaire of Spinal Cord Injury Care in Rehabilitation Facilities	
**Question**	**Answer**
**REHAB CARE – DISCHARGE INFORMATION**	
48. How many patients were discharged to the following destinations after rehab care in a year?	Please enter separately for patients with traumatic SCI and those with non-traumatic SCI:<br>• Home<br>• Another inpatient rehab centre<br>• Acute hospital<br>• Long-term care centre<br>• Assisted living centre<br>• Group living centre<br>• Transitional centre<br>• In-hospital death<br>• Other (please specify)
49. What are the common barriers to discharge from rehab?	
50. A) Do you have facilities available where patients can live while waiting for home renovations?<br>B) If yes, please list and describe criteria for using these facilities.	A) Yes/No<br>B)
**REHAB CARE – STANDARDS OF CARE**	
51. A) Is the practice of care standardized in your country/region (e.g. accreditation, clinical practice guideline/evidence-based protocols)?<br>B) If yes, provide brief details of standards available.<br>C) If yes, are the standards mandatory to follow?<br>D) If not, does your centre follow the standards?	A) Yes/No<br>B)<br>C) Yes/No<br>D) Yes/No
